# Dataset of clinical cases, images, image labels and captions from open access case reports from PubMed Central (1990–2023)

**DOI:** 10.1016/j.dib.2023.110008

**Published:** 2023-12-23

**Authors:** Mauro Andrés Nievas Offidani, Claudio Augusto Delrieux

**Affiliations:** Department of Electrical and Computer Engineering, National University of the South, Avda. Alem 1253 - Body A - 1st Floor, B8000CPB Bahía Blanca, Argentina

**Keywords:** Multimodal, Medical, Healthcare, Radiology, Pathology, Text

## Abstract

This paper details the acquisition, structure and preprocessing of the MultiCaRe Dataset, a multimodal case report dataset which contains data from 75,382 open access PubMed Central articles spanning the period from 1990 to 2023. The dataset includes 96,428 clinical cases, 135,596 images, and their corresponding labels and captions. Data extraction was performed using different APIs and packages such as Biopython, requests, Beautifulsoup, BioC API for PMC and EuropePMC RESTful API. Image labels were created based on the contents of their corresponding captions, by using Spark NLP for Healthcare and manual annotations. Images were preprocessed with OpenCV in order to remove borders and split figures containing multiple images, data were analyzed and described, and a subset was randomly selected for quality assessment. The dataset's structure allows for seamless integration of different types of data, making it a valuable resource for training or fine-tuning medical language, computer vision or multi-modal models.

Specifications Table

**Table:** 

Subject	Health and medical sciences
Specific subject area	Medical Imaging; Radiography and Radiology; Pathology and Medical Technology
Data format	Raw, Analyzed, Filtered
Type of data	Image, Text
Data collection	- Clinical cases, article metadata and captions were collected using Biopython, requests, Beautifulsoup and BioC API for PMC - Images were collected using EuropePMC RESTful API - Image labels were created using manual annotations and Spark NLP for Healthcare Details about how the dataset was created can be found in this repository: https://github.com/mauro-nievoff/MultiCaRe_Dataset/tree/main
Data source location	Department of Electrical and Computer Engineering, National University of the South (Bahía Blanca, Argentina)
Data accessibility	Repository name: Zenodo Data identification number: 10.5281/zenodo.10079370 Direct URL to data: https://zenodo.org/records/10079370

## 1 Value of the Data

• The dataset contains multi-modal data from over 75,000 open access and de-identified case report articles, including metadata, clinical cases, image captions and more than 130,000 images. Almost 100,000 patients and almost 400,000 medical doctors and researchers were involved in the creation of the articles included in this dataset.
• The dataset contains images and cases from different medical specialties, including oncology, cardiology, surgery and pathology.
• The dataset can be used to train or fine-tune machine learning models, medical language, computer vision or multi-modal models. The structure of the dataset allows to easily map images with their corresponding article metadata, clinical case, captions and image labels.

## 2 Background

The motivation behind the compilation of this dataset lies in addressing the scarcity of publicly available multimodal datasets with clinical data by leveraging the wealth of valuable information included in case report articles.

## 3 Data Description

### 3.1 Dataset structure

The dataset contains the following files or folders (8.76 GB in total) [1]:

• metadata.parquet: It contains the metadata for each case report article in JSON format, including title, author, journal, journal details, year, DOI, PMID, PMCID, MeSH terms, major MeSH terms, keywords, link, license type and amount of clinical cases.
• cases.parquet: It contains article ID, case ID, case text (in English), and age and gender of the patient.
• case_images.parquet: it contains article ID, case ID, image ID, file name, tag, captions and text references.
• abstracts.parquet: It contains article ID and abstract.
• image folders: Folder names correspond to the first 4 characters from the article PMCID, and subfolders named correspond to the first 6 characters (e.g. the image PMC10000323_jbsr-107–1–3012-g3_undivided_1_1.jpg is found in the PMC100 folder, which is found in the PMC1 folder). Images are not always exactly the same as the raw files included in the original articles, because any single raw file containing multiple images was split and borders were removed during image preprocessing.
• captions_and_labels.csv: Each row corresponds to one image file from the dataset (any raw caption containing multiple captions was split before creating this file). The CSV file contains file ID, file name, raw image ID, patient ID, image license, caption, extracted chunks and image labels (which are included in different columns).
• data_dictionary.csv: It contains file names, data fields and their explanations. Please refer to this file for more specific details about the file contents.

### 3.2 Data analysis

**Fig. 1:**
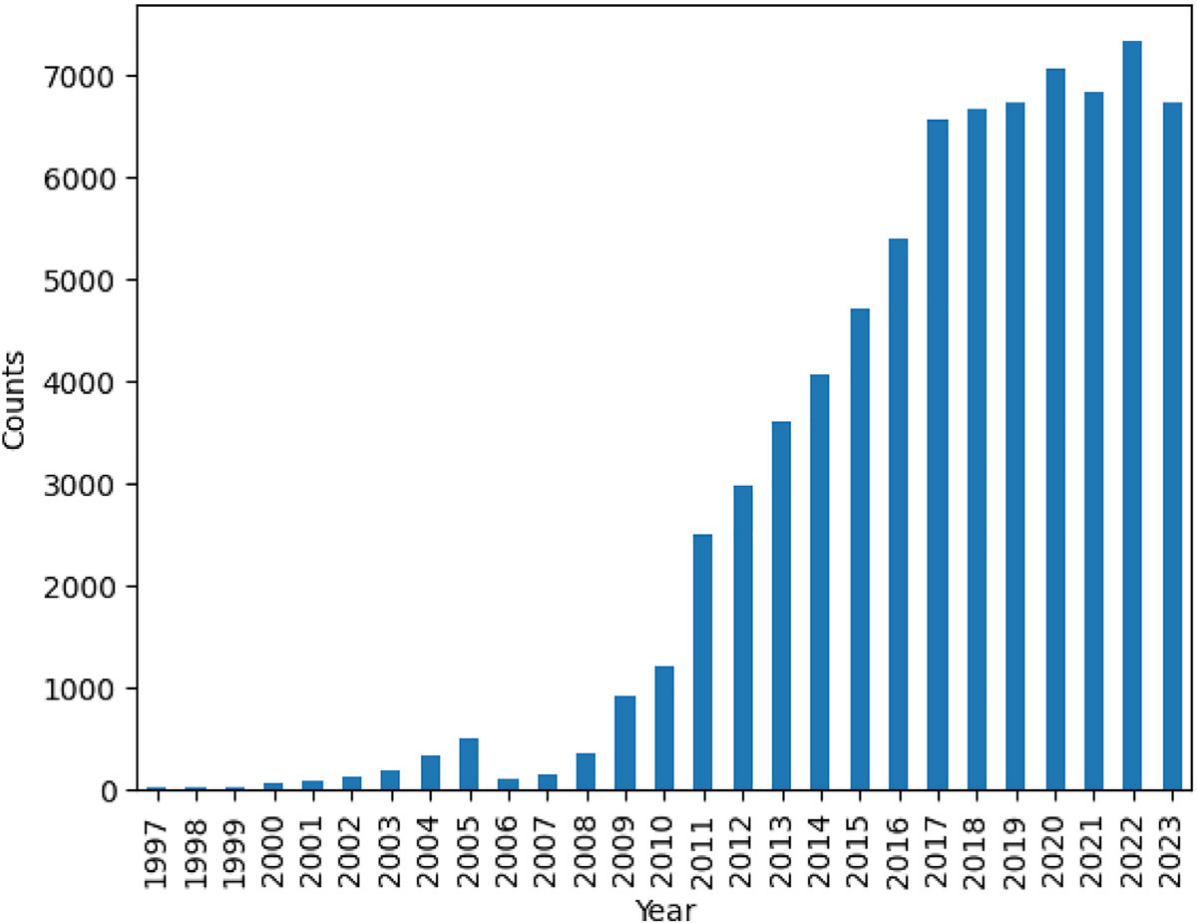
Amount of case report articles per year.

**Fig. 2:**
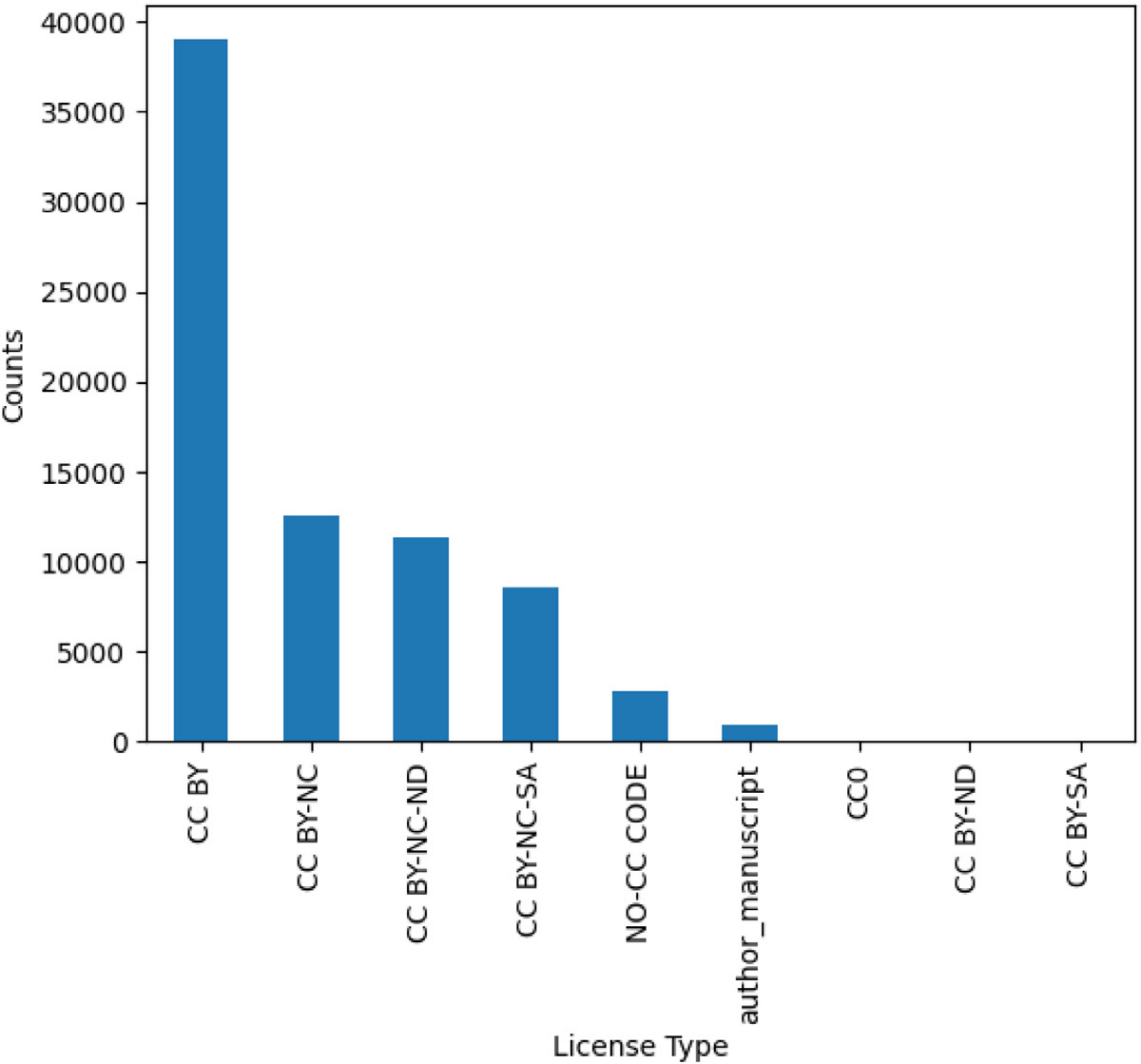
Amount of articles per license type.

A total of 75,382 case report articles are included in this dataset, describing 96,428 clinical cases. The articles were published by 387,962 authors in 2468 journals between January 1990 and August 2023 (see temporal distribution in Fig. 1). All the articles are open access, and they have different license types (see Fig. 2). There is a mean of 1.3 patients per article (85 % of them describe only one patient). Each clinical case has an average of around 400 words.

**Fig. 3:**
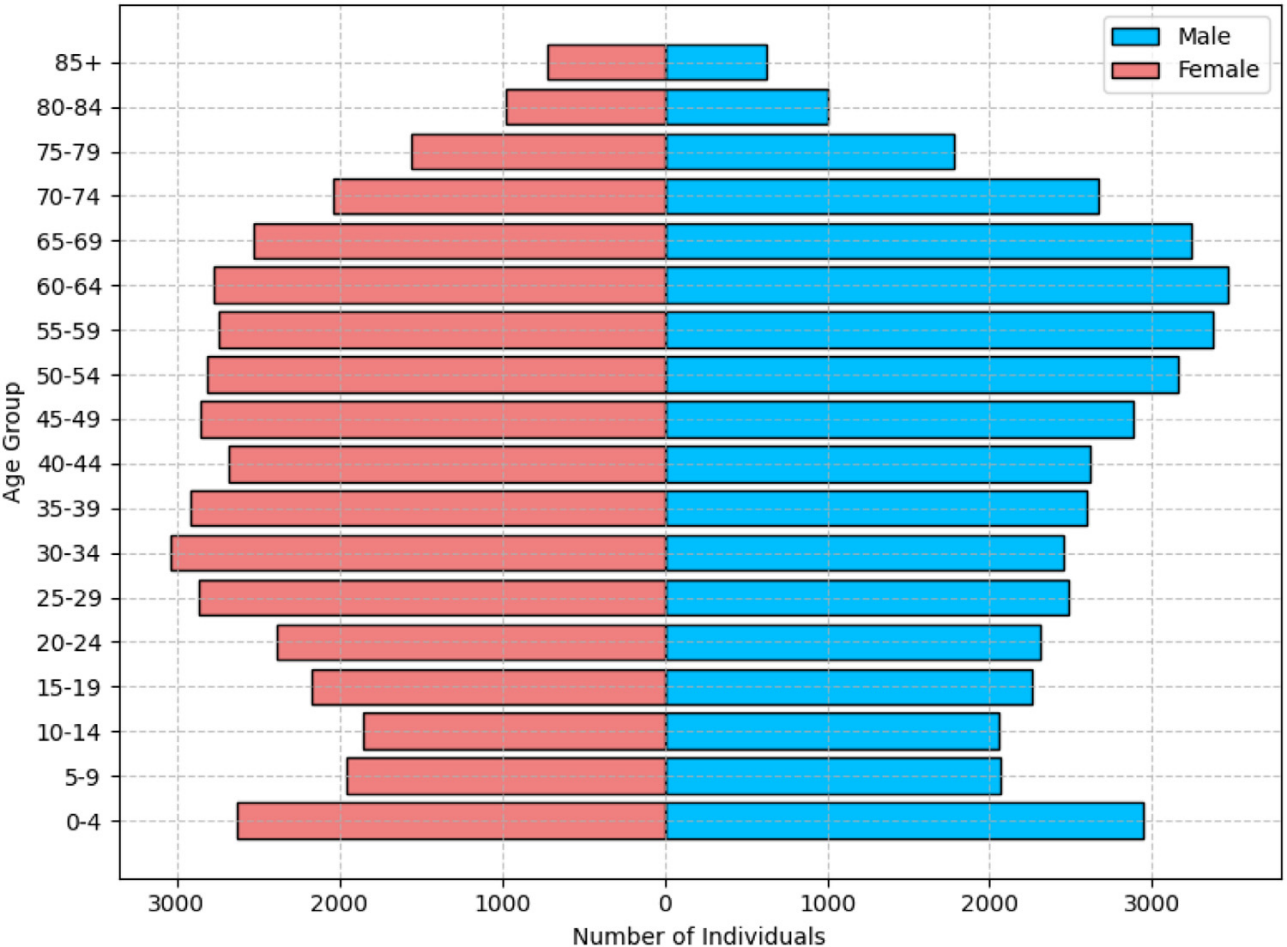
Population Pyramid of the clinical cases included in the dataset.

The dataset includes 43,867 female patients, 46,588 male patients, 96 transgender patients and 5877 patients with unknown gender. The age mean of the whole dataset is 41.5 years-old (see Fig. 3 for more detail on the demographic distribution).

**Fig. 4:**
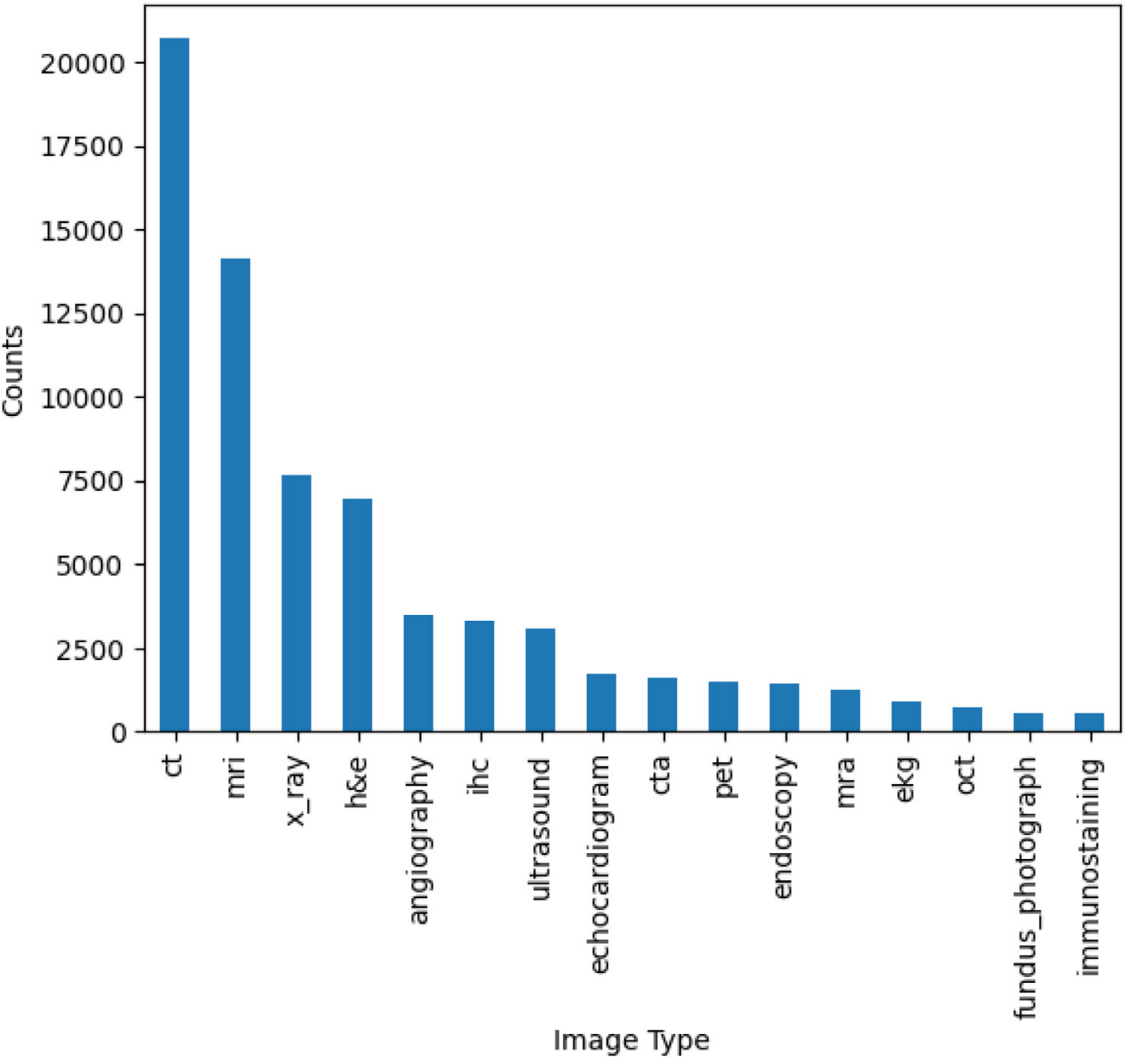
Amount of images per type. ct: computed tomography, mri: magnetic resonance imaging, h&e: hematoxylin and eosin, ihc: immunohistochemistry, cta: computed tomography angiography, pet: positron emission tomography, mra: magnetic resonance angiography, ekg: electrocardiogram, oct: optical coherence tomography.

This dataset has in total 135,596 images. A detail on the image types can be found in Fig. 4 (considering the labels from the image_type and pathology_test columns from captions_and_labels.csv). The most common image types and their corresponding most frequent associated anatomical site labels are: CT scan (brain, lung, chest, bone, spine, abdomen), MRI (brain, spine), X-rays (chest, bone, lung), hematoxylin and eosin stain, angiography, immunohistochemistry and ultrasound (abdomen, liver).

### 3.3 Differences between case report data and real clinical data

Clinical cases are not medical records. Their text can be annotated and used, for example, to train Natural Language Processing models to extract information from medical records, but it's important to mention some differences between these two types of texts:

• Normal cases: Patients that don't have any significant clinical problem will not be included in a case report dataset, but normal cases are usually found in medical record datasets (e.g. routine checkups or screening visits).
• Rare cases: These may be over-represented in a case report dataset, and this can be considered as something positive because it adds more variability and diversity to the dataset.
• Irrelevant information: Phrases like 'patient denies any chest pain' are often found in medical records. In contrast, case reports usually do not mention symptoms that are absent and normal test results may not be included either.
• Text quality: Although text quality in medical records is high, when it comes to case reports it is much higher, as they undergo a rigorous review process before being published in a medical journal.
• Personal information: Case reports are intended to be shared publicly, so they do not include any personal information of the patient. Medical records, however, are not de-identified because they are confidential documents maintained within a healthcare institution.

When it comes to images, data from case reports also differ from the ones present in health record datasets: images from case reports are much smaller in size, they may contain burned drawings (such as arrows, circles or asterisks) and they do not contain any personal information of the patient.

## 4 Experimental Design, Materials and Methods

### 4.1 Pubmed search

The query string was created considering the following conditions: either the “Publication Type” field of the article should be “case report” or its “Title/Abstract” field should contain relevant mentions such as “clinical case”, “case report” or “case series”; only full free-text articles should be retrieved (by using the “ffrft” filter); articles tagged as related to “animals” should be filtered out; and only articles after 1990 should be included. The language was not used as a filter (articles in a language other than English were filtered out during the process of data extraction due to the strings used for regex matching and other tasks).

Then, Biopython was used to get the PMIDs of all the articles that matched that query [2]. As there is a limit in the amount of articles that can be retrieved in each query (9999), the query was split into multiple queries using different time ranges at different scales (year, month or day). When a particular day contained more than 9999 articles, the articles over that limit were lost. Biopython was also used to map each PMID to its corresponding PMCID, which was then used to get the article metadata and contents through different APIs from PMC and EuropePMC. The contents were not queried using such APIs directly because PubMed's search engine was found to be more useful than PMC's.

### 4.2 Article text content

The article metadata and abstracts were retrieved using the packages requests [3] and BeautifulSoup [4]. For the rest of the text content (including image captions and file names), BioC API for PMC was used [5]. Case related text parts were recognized considering HTML tags, headers, or paragraph contents (mention of ages, which are very common at the beginning of clinical cases). Mentions of demographic information (age or gender-specific words such as “lady” or “boy”) were used to identify if multiple cases were present in the same article. The actual age and gender of the patient were extracted by using regex patterns. Images were matched to their corresponding cases by identifying figure mentions in text, and sentences including such mentions were extracted as text references and assigned to their corresponding image in the case_images.parquet file.

### 4.3 Turning captions into image labels

Each image was assigned labels based on the content of its corresponding caption. It's important to mention that this method of label assignment may result in many false negatives: an image labelled as “ct” can be considered to be a CT scan, but an image without a “ct” label can also be a CT scan if the corresponding caption did not mention the image type (it can only be considered not to be a CT scan if a mutually exclusive label is present, such as “mri”).

In some cases, a single caption may refer to multiple images from a single article figure, which makes caption splitting necessary. To understand this, let's consider the following example caption: *“Brain CT scan. There is a mass in the frontal lobe (A-C) and an intracerebral haemorrhage in the right parietotemporal lobe (C and D)”*. Preprocessing functions were created using regex, in order to split the caption into four pieces (A, B, C and D). The initial string (“Brain CT scan”) was assigned to all the pieces, “There is a mass in the frontal lobe” was assigned to piece A, B and C, and “and an intracerebral haemorrhage in the right parietotemporal lobe” was assigned to C and D.

The extraction of relevant data from captions was done using contextual parsers included in the library Spark NLP for Healthcare from John Snow Labs [6]. Contextual parsers extract data based on dictionaries (in this case, csv files with label names and strings chunks of text that should be extracted using those labels). These csv dictionaries were created by manually annotating all the n-grams of the whole caption dataset for different values of n (unless their frequency in the dataset was too low). When creating the pipeline, longer n-grams were prioritized over shorter ones (so that, for example, the text “ct scan” was extracted altogether, instead of extracting “ct” alone). The taxonomy used to annotate the n-grams included labels related to the image type (such as Imaging_Test, Pathology_Test, Image_Technique), image findings (such as Image_Finding, Problem, EKG_Finding), anatomy (such as Site or Laterality) or negation (Assertion_Absent, to extract words such as “no” or “without”).

The extractions from contextual parsers were normalized using a normalization dictionary (in order to map, for instance, “CT”, “CT scan” and “tomography” to a common image label called “ct”). This dictionary was created manually. The normalized extractions for each caption can be found in the last columns of the captions_and_labels.csv file (pathology_test, image_type, image_technique, image_view, site, laterality, finding and negation).

### 4.4 Image preprocessing

**Fig. 5:**
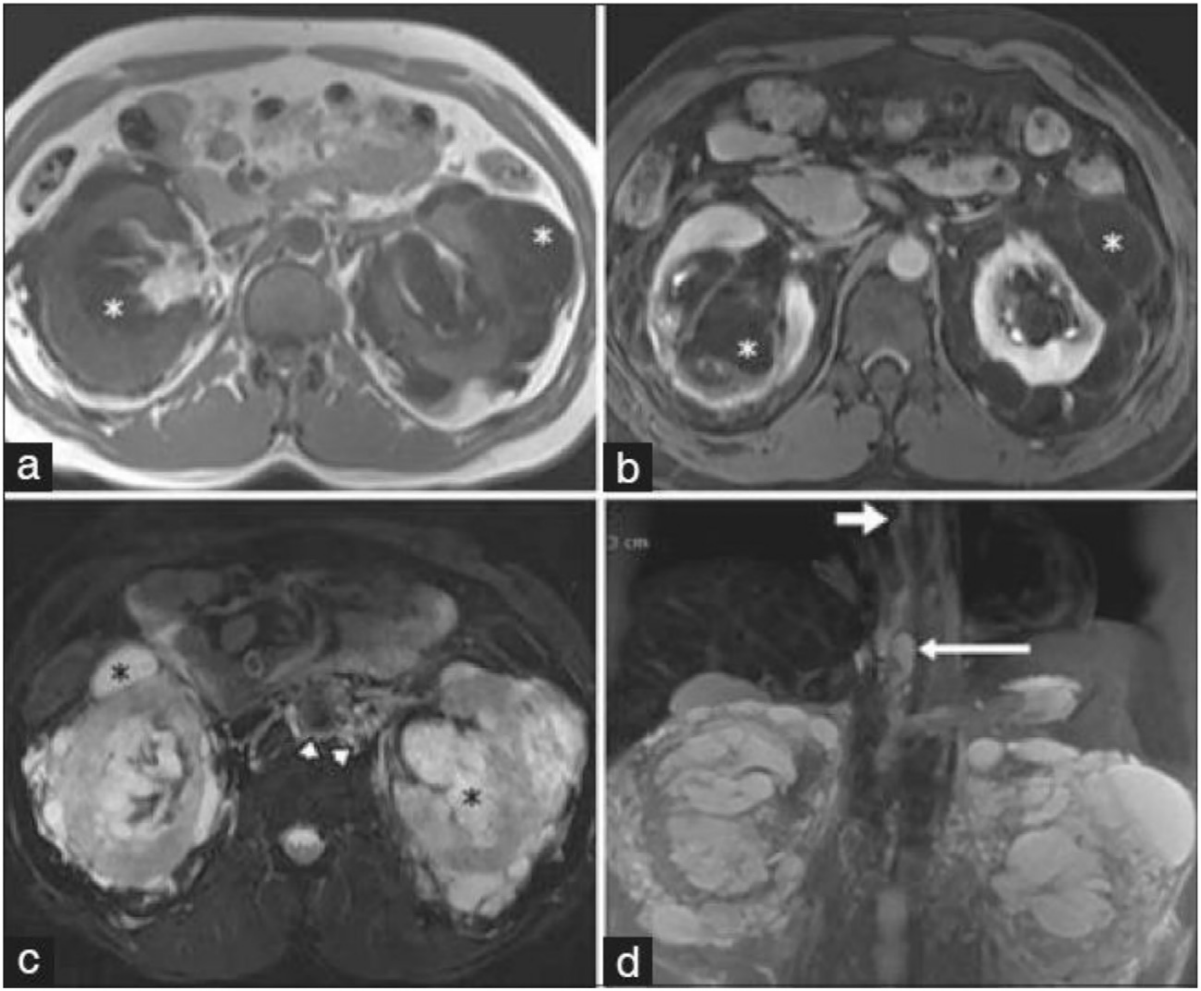
Example of an article figure with multiple images (taken from a case report included in the dataset [9]).

Article figures were downloaded using EuropePMC RESTful API [7]. Figures that are not mentioned in the content of a clinical case were not included in the dataset (this happens, for example, if they are mentioned in the introduction of the article). It is relatively common to find figures that include more than one image, such as Fig. 5. OpenCV was used during file preprocessing in order to split such cases into individual images, and also to remove any image border that may be present [8].

Image preprocessing consisted of these steps:

1. Edge Detection: First the image was blurred using a Gaussian filter, and then a Canny edge detector was applied (see an example of outcome in Fig. 6).

**Fig. 6:**
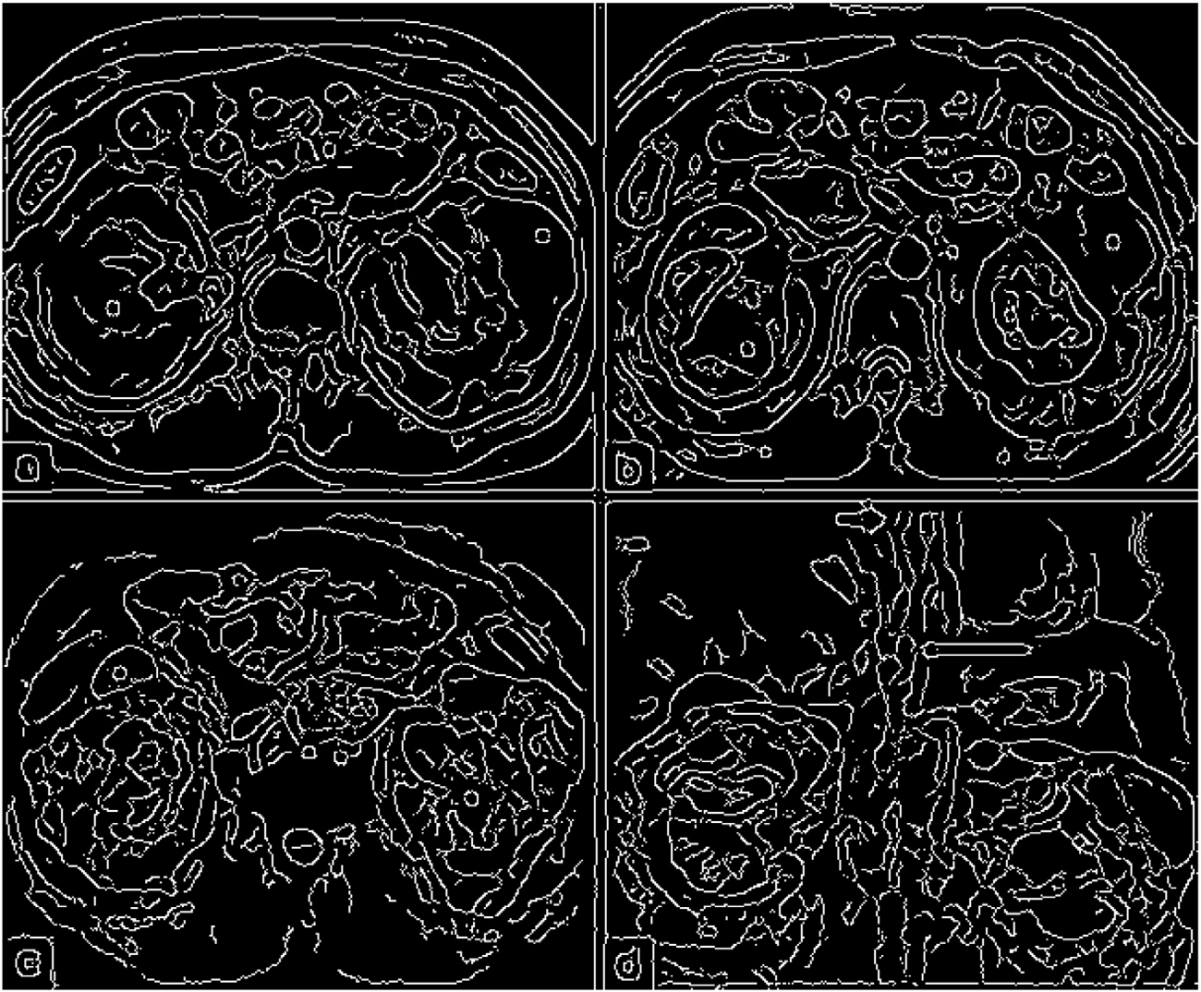
Edge detection for the image from Fig. 5.
2. Image Limit Detection: Rows or columns of pixels that contain a low amount of edges are considered to be image limits.
3. Image Slicing: Images are split considering the limits that were detected (see example outcome in Fig. 7). Any resulting image smaller than a certain threshold is discarded.

**Fig. 7:**
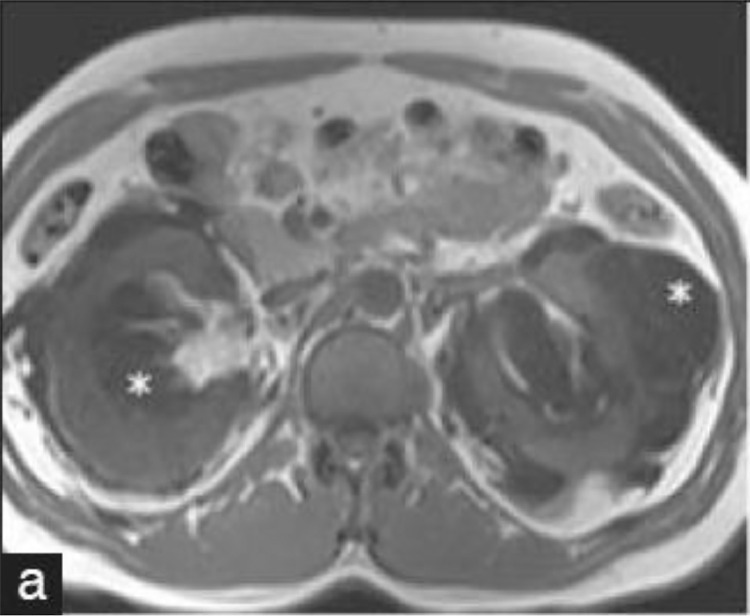
Example of one of the outcome images after Fig. 5 is split.
4. Order Sorting: Images are sorted considering their position in the original figure, left to right, top to bottom (so that, for example, the second image is always the one to the right from the first image).

The results from this image preprocessing were cross-checked with the ones from caption splitting as a way to improve the quality of the dataset. This dataset includes only the images that were split into the same number of pieces as their corresponding captions. This means that if a figure was split into 2 images and its corresponding caption was split into 3 pieces, these images were discarded.

## 5 Limitations

### 5.1 Data quality

Textual data from 100 case reports were reviewed and these were the results:

• Data without issues: 84 %
• Wrong article type (not case report): 3 %
• At least one wrong demographic extraction: 4 %
• Extra split (the same patient split into more than one case): 3 %
• Missing split (at least two patient presentations included in the same case): 3 %
• Wrong article content included in the text of a case (such as introduction): 3 %

A total of 153 images (corresponding to 100 article figures) were reviewed and these were the results:

• Data without issues: 86 %
• Figures containing multiple images were not split into single images (not detected because of wrong captions): 4 %
• Wrong image order: 2 %
• Relevant part of the image was removed: 2 %
• Wrong label assignment: 2 %
• Wrong patient assignment: 1 %

## Ethics Statement

The authors have read the ethical requirements for publication in Data in Brief. The current work does not involve human subjects, animal experiments, or any data collected from social media platforms.

## CRediT authorship contribution statement

**Mauro Andrés Nievas Offidani:** Conceptualization, Data curation, Writing – original draft. **Claudio Augusto Delrieux:** Writing – review & editing.

## Data Availability

MultiCaRe Dataset (Original data) (Zenodo)
